# *QuickStats:* Age-Adjusted Death Rates* from Unintentional Falls Among Adults Aged ≥65 Years,^†^ by Race/Ethnicity — National Vital Statistics System, United States, 2001–2016

**DOI:** 10.15585/mmwr.mm6718a7

**Published:** 2018-05-11

**Figure:**
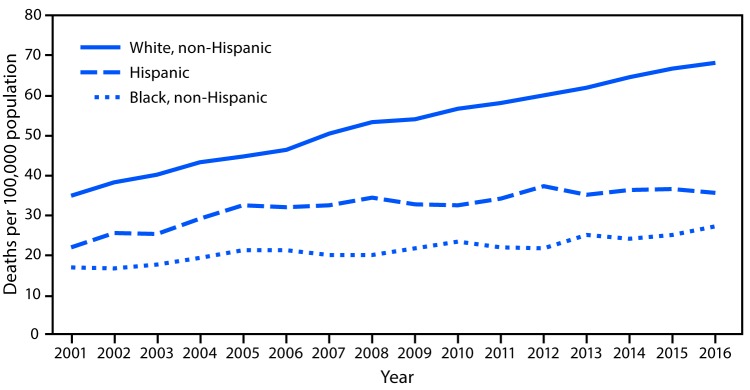
During 2001–2016, the age-adjusted death rate for unintentional falls for non-Hispanic white adults aged ≥65 approximately doubled, increasing from 34.9 deaths per 100,000 to 68.7. In that period, the death rate for Hispanic adults increased from 21.9 to 35.7, and the rate for non-Hispanic black adults rose from 16.8 to 27.1. Throughout the period, the death rate from falls for non-Hispanic white adults was 1.4 to 1.9 times the rate for Hispanic adults and 2.1 to 2.8 times the rate for non-Hispanic black adults.

**Source:** National Vital Statistics System, 2001–2016. https://wonder.cdc.gov/ucd-icd10.html.

For more information on this topic, CDC recommends the following link: https://www.cdc.gov/homeandrecreationalsafety/falls/index.html.

